# Left ventricular systolic dysfunction predicts clinical prognosis in patients with acute ischemic stroke after intravenous thrombolysis

**DOI:** 10.18632/aging.205786

**Published:** 2024-05-02

**Authors:** Chi Zhang, Jun-Cang Wu, Zheng Tan, Xiao-Lu He, Fei Li, Long Wang, Yu Wang

**Affiliations:** 1 Department of Neurology, The First Affiliated Hospital of Anhui Medical University, Hefei 230011, Anhui, China; 2 Department of Neurology, Hefei Hospital Affiliated to Anhui Medical University (The Second People’s Hospital of Hefei), Hefei 230011, Anhui, China

**Keywords:** acute ischemic stroke, left ventricular systolic dysfunction, left ventricular ejection fraction, intravenous thrombolysis, outcome

## Abstract

Background: Although intravenous recombinant tissue plasminogen activator (rt-PA) thrombolysis is the most effective early treatment for acute ischemic stroke (AIS), outcomes vary greatly among patients. Left ventricular systolic dysfunction (LVSD) is prone to distant organ ischemia and may be a predictor for poor prognosis in AIS patients undergoing intravenous thrombolysis (IVT). Our aim was to investigate the predictivity of LVSD diagnosis (as measured by left ventricular ejection fraction (LVEF)) on 90-day clinical outcomes in AIS patients undergoing thrombolysis.

Methods: The current prospective cohort study continuously enrolled 273 AIS patients from the National Stroke Prevention and Treatment Engineering Management Special Database who underwent IVT and completed echocardiography within 24 h of admission between 2021 and 2023. LVSD was examined by evaluation of the echocardiographic LVEF values using Simpson’s biplane method of discs in line with international guidelines, and defined as a LVEF value < 50%. Multivariable ordinal logistic regression model was performed to analyze the association between LVEF and functional outcome at 3 months. Restricted cubic spline (RCS) was used to examine the shape of the dose-response association between reduced LVEF and poor functional outcomes. Subgroup analysis was also employed to further verify the reliability and practicability of the results.

Results: Baseline data analysis showed LVSD patients had more comorbidities including on multivariate analyses, LVSD (OR 2.78, 95% CI 1.23 to 6.24, P=0.014), pre-existing diabetes mellitus (OR 2.08, 95% CI 1.11 to 3.90, P=0.023) and NIHSS on arrival (OR 1.31, 95% CI 1.21 to 1.49, P<0.001) were independent predictors of poor functional outcomes (mRS ≥ 3) at 3 months. Multivariable-adjusted spline regression indicated a linear dose-response association between LVEF after IVT and poor functional outcomes (p for linearity < 0.001), with the optimal cutoff values of LVEF being 0.48.

Conclusions: Our finding indicated that AIS patients with LVSD after IVT had poorer outcomes, suggesting the need to monitor and optimize LVEF in stroke management.

## INTRODUCTION

The latest epidemiological data showed that stroke is the leading cause of death and disability globally among the 18 neurological disorders [1]. Of all stroke types, ischemic stroke (IS) accounts for almost 80% [2]. Despite intravascular thrombectomy (ET) has broad application prospects in the new era of intravascular therapy, intravenous thrombolysis (IVT) remains the preferred and fundamental treatment for acute ischemic stroke (AIS) within 4.5 h of symptom onset as recommended by the guidelines [3, 4]. Nevertheless, higher risks of hemorrhagic transformation (HT) and neuroinflammation are related to IVT treatment due to the ischemia-reperfusion injury, rt-PA toxicity, and comorbidities, which may lead to worse functional outcomes in AIS patients, thus rendering this life-saving therapy a double-edged sword [5, 6].

Left ventricular systolic dysfunction (LVSD) has been reported as an important clinical predictor of higher morbidity and mortality in AIS patients [7, 8]. Left ventricular ejection fraction (LVEF) is a common parameter for the evaluation of left ventricular systolic function, and is widely used in clinical diagnosis and experimental research [9–11]. Previous studies prior to IVT or ET have indicated that reduced LVEF was associated with higher NIHSS scores at admission, poorer functional outcomes and higher in-hospital mortality rates [8, 12]. LVSD may involve in the pathophysiology of AIS through different mechanisms. It has been suggested that LVSD may predispose to *in situ* thrombosis as well as proximal thrombosis formation, leading to intracranial vascular occlusion after detachment which decreases global cerebral blood flow. In addition, LVSD causes a state of global cerebral hypoperfusion with decreased brain blood flow and cerebrovascular reactivity [13, 14], which contributes to a higher risk of ischemic insult.

Although LVEF has been routinely investigated during hospitalization in AIS patients, its impact on clinical outcomes remains controversial due to the lack of consistent LVEF classification standards. Moreover, few studies focused on the association between LVSD and functional prognosis in AIS patients undergoing IVT. Therefore, in current prospective cohort registry for AIS undergoing IVT, we aimed to investigate the relationship between LVSD and clinical outcomes and to explore the optimal nodes for predicting poor prognosis with reduced LVEF.

## MATERIALS AND METHODS

### Study population

**Figure 1:**
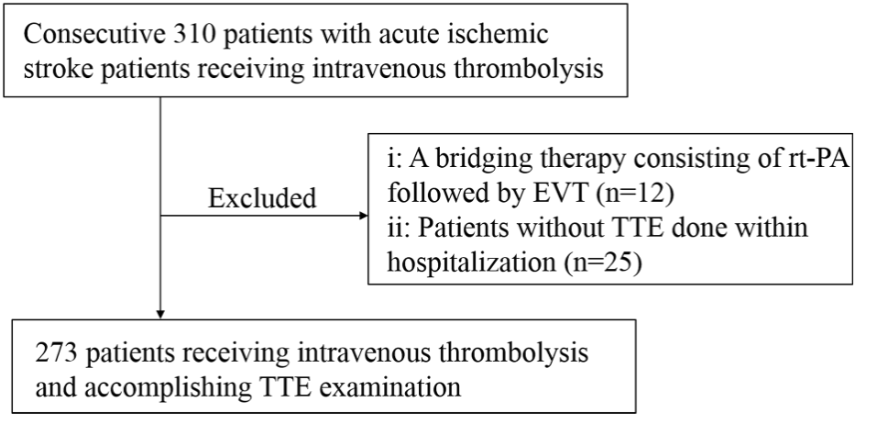
**Flowchart of study population selection.** rt-PA, recombinant tissue plasminogen activator; EVT, Endovascular therapy; TTE, Transthoracic echocardiography.

From June 2021 to May 2023, we prospectively collected a total of 310 patients treated with intravenous rt-PA (dose of 0.9 mg/kg, maximum 90mg) within 4.5 h of AIS onset in the National Stroke Prevention and Treatment Engineering Management Special Database. Of these, twelve patients who underwent intravenous thrombolysis combined with ET, and twenty-five patients with lack of LVEF data were excluded. Ultimately, 273 patients were eligible for inclusion in the final analysis (Figure 1).

### Data collection

Data regarding the demographic and clinical characteristics, age, sex, blood pressure, onset-to-needle time (ONT), stroke subtype, lifestyle risk factors (smoking and drinking alcohol), past medical history (hypertension, diabetes mellitus, hyperlipemia, previous stroke, coronary heart disease and atrial fibrillation) were collected from electronic medical records. ONT is further subdivided into less than 3 h and 3 to 4.5 h. Stroke subtype was determined based on the TOAST criteria [15]. The severity of AIS was assessed by professional clinicians via the National Institutes of Health Stroke Scale (NIHSS) score at admission and 24 h after IVT [16]. The modified Rankin Scale (mRS) score with seven levels was used to assess neurological functional outcomes at the 3-month follow-up [17].

### Clinical measurement and definition of LVSD

LVSD was measured by the evaluation of the LVEF on transthoracic echocardiography (TTE) using Simpson’s biplane method of discs in line with international guidelines.

This procedure was based on the tracking of the central intimal boundary in both the apical four- and two-chamber views at the end of systole and diastole performed by a trained echocardiologist. Echocardiography and data extraction were completed within 24 h after admission. LVSD was defined as LVEF <50% according to previous descriptions.

### Clinical outcome

The severity of AIS was assessed by well-trained neurologists at baseline and 24 h after rt-PA thrombolysis using the National Institutes of Health Stroke Scale (NIHSS) score [16]. Early neurological deterioration (END) was defined as an increment in the total NIHSS score of ≥ 4 points within 24 h after thrombolysis [18]. Complications of rt-PA intravenous thrombolysis, including symptomatic intracranial hemorrhage (sICH) and hemorrhagic transformation (HT) were evaluated in accordance with the European Cooperative Acute Stroke Study II (ECASS-II) criteria [19]. In brief, sICH was defined as a newly occurring hyper-density around the infarction on CT with neurological deterioration (NIHSS score ≥4) within 24 hours after intravenous thrombolysis. The Modified Rankin Scale (mRS) was utilized to assess functional outcomes after 90 days, with a score ≤ 2 representing a good outcome, whereas a score of 3~6 represents a poor outcome [17]. The primary outcome was deemed as a good functional outcome with a 90-day mRS score of 0~2, while the secondary outcomes evaluated were END, sICH, and HT.

### Statistical analysis

Statistical analyses were conducted using SPSS version 17.0 software (SPSS Inc., Chicago, IL, USA) and R version 4.1.0 (R Foundation for Statistical Computing, Vienna, Austria). Continuous variables conforming to normal distribution were shown as mean ± standard deviation (SD) and compared using Student’s t-test or One-way ANOVA. Categorical variables were presented as percentages and analyzed by using Chi-square test. After adjusting for confounding factors, multivariable ordinal logistic regression model was used to analyze the association between LVEF and functional outcome at three months based on variables with a p-value < 0.05 in univariate analyses. A nonlinear logistic regression with restricted cubic spline (RCS) was used to examine the shape of the association between LVEF and primary outcome with four knots (defined at the 5th, 35th, 65th, and 95th percentiles). Subgroup analysis was also performed to further verify the reliability and practicability of the results and the multiplicative interaction terms were tested by the likelihood ratio test. A 2-tailed p-value < 0.05 was considered as the level for statistical significance.

## RESULTS

### Baseline characteristics of study samples

As shown in Table 1, a total of 273 AIS patients (169 men and 104 women) were finally included after screening, with a mean age of 69.2 ±12.5 years, then divided into two groups (LVEF ≥50% group and LVEF<50% group). LVSD was observed in 41 (15.0%) patients. There were no significant differences in age, sex, stroke risk factors (CHD, smoking, and drinking), time from onset to intravenous thrombolysis (ONT), as well as sICH (p>0.05). However, hyperlipidemia, DM, coronary heart disease, and initial higher NIHSS scores were more commonly found in LVSD patients compared to non-LVSD patients (p<0.05).

**Table 1:** Baseline characteristics of patients stratified by the degree of LVEF.

**Variable**	**Total (n=273)**	**LVEF ≥ 50% (n=232)**	**LVEF < 50% (n=41)**	***P*-value**
Demographic data				
Age, years mean (SD)	69.2±12.5	68.8±12.8	71.7±10.5	0.082
Male [n(%)]	169 (61.9)	146 (63.0)	23 (56.1)	0.406
Stroke risk factors [n(%)]				
Hypertension	136 (58.6)	108 (46.6)	26 (63.4)	0.034
Diabetes	66 (28.4)	51 (22.0)	15 (36.6)	0.044
Hyperlipidemia	110 (40.3)	97 (41.8)	18 (43.9)	0.803
CHD	100 (43.1)	83 (35.8)	19 (46.3)	0.197
Smoking	90 (33.0)	81 (35.0)	9 (22.0)	0.104
Drinking	84 (30.8)	73 (31.5)	11 (26.8)	0.553
History of stroke or TIA [n(%)]	38 (13.9)	32 (13.8)	6 (14.6)	
Stroke etiology, TOAST [n(%)]				0.502
Large artery atherosclerosis	167 (60.9)	144 (61.8)	23 (56.1)	
Cardioembolism	13 (4.7)	9 (3.9)	4 (9.8)	
Small artery occlusion	80 (29.2)	69 (29.6)	11 (26.8)	
Other determined	10 (3.6)	8 (3.4)	2 (4.9)	
Undetermined	4 (1.5)	3 (1.3)	1 (2.4)	
ONT [n(%)]				0.698
< 3 h	186 (68.1)	157 (67.7)	29 (70.7)	
3 to 4.5 h	87 (31.9)	75 (32.3)	12 (29.3)	
Stroke evaluation				
NIHSS on arrival, median (IQR)	6 (5, 8)	6 (4, 8)	8 (6, 10)	<0.001
NIHSS at 24 h, median (IQR)	4 (2, 7)	4 (2, 6)	8 (5, 10)	<0.001
Outcomes [n(%)]				
mRS (0~2) at 90 days	154 (56.4)	143 (61.6)	11 (26.8)	0.003
sICH or HT	8 (2.9)	5 (2.2)	3 (7.3)	0.071
END	11 (4.0)	6 (2.6)	5 (12.2)	0.004

### LVSD correlations with clinical outcome

**Figure 2:**
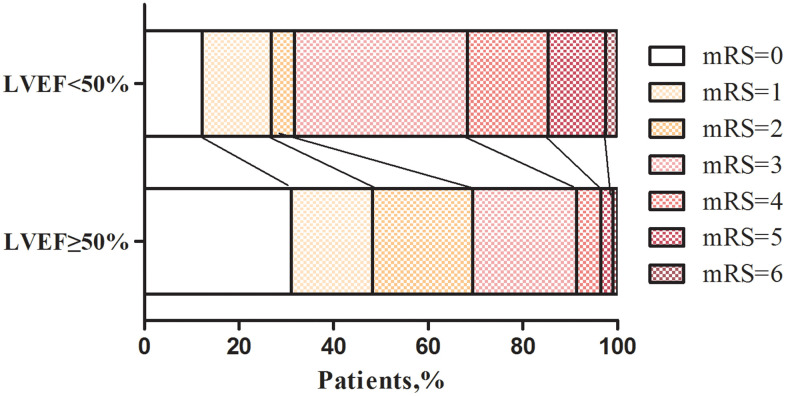
**Distribution of mRS scores at three months according to LVEF levels after IVT in AIS patients.** LVEF, left ventricular ejection function.

Fewer LVSD patients achieved a good functional outcome at 90 days (mRS≤2), however, there was no significant difference in the proportion of patients with sICH or HT compared with the non-LVSD group. By analyzing the distribution of mRS between the two groups, the relationship between LVEF levels and prognosis emerged. LVSD patients had significantly worse mRS scores after IVT in comparison with LVEF ≥ 50% patients (Figure 2). After adjusting for age, gender, and other conventional confounders, multivariate logistic regression analysis showed that higher NIHSS score (OR 1.08, 95% CI 1.04 to 1.11; p<0.001), pre-existing diabetes (OR 2.08, 95% CI 1.11 to 3.90; p=0.023), and presence of LVSD (OR 2.78, 95% CI 1.23 to 6.26; p=0.014) were associated with the 90-day poor functional outcomes (Table 2).

**Table 2:** Univariate and multivariate analyses of predictors of poor functional outcomes in AIS patients.

**Variable**	**Univariate analysis**				**Multivariate analysis**	
	**mRS (0~2)**	**mRS (3~6)**	**OR (95%CI)**	**P-value**	**Adjusted OR (95%CI)**	**P-value**
Age (years), mean (SD)	68.3±12.3	70.4±12.8	1.01 (0.99, 1.03)	0.181	1.00 (0.98, 1.02)	0.922
Male [n(%)]	101 (59.8)	68 (40.2)	0.70 (0.43, 1.15)	0.155	0.80 (0.45, 1.42)	0.453
Hypertension, [n(%)]	66 (48.5)	70 (51.5)	1.40 (0.86, 2.26)	0.173	1.07 (0.62, 1.84)	0.814
Diabetes, [n(%)]	24 (36.4)	42 (63.6)	2.96 (1.66, 5.25)	<0.001	2.08 (1.11, 3.90)	0.023
Hyperlipidemia, [n(%)]	71 (64.5)	39 (35.5)	0.69 (0.42, 1.12)	0.130	0.66 (0.38, 1.16)	0.145
CHD, [n(%)]	44 (44.0)	56 (56.0)	1.25 (0.76, 2.05)	0.372	1.07 (0.61, 1.87)	0.822
History of stroke or TIA, [n(%)]	18 (47.4)	20 (52.6)	1.44 (0.72, 2.88)	0.308	1.15 (0.52, 2.54)	0.732
IVT 3h [n(%)]	45 (51.7)	42 (48.3)	1.18 (0.70, 1.98)	0.528	1.32 (0.74, 2.36)	0.347
NIHSS on arrival, median (IQR)	6 (4, 7)	7 (6, 9)	1.40 (1.24, 1.58)	<0.001	1.31 (1.21, 1.49)	<0.001
Reduced LVEF, [n(%)]	11 (26.8)	30 (73.2)	4.38 (2.09, 9.18)	<0.001	2.78 (1.23, 6.26)	0.014
LVEF by category, [n(%)]						
LVEF (≥50%)	143 (52.3)	89 (32.6)	(Reference)			
LVEF (41~49%)	10 (3.7)	19 (7.0)	3.21 (1.44, 7.18)	0.009		
LVEF (≤40%)	1 (0.4)	11 (4.0)	16.07 (2.02, 127.66)	0.004		

LVEF, Left ventricular ejection fraction; NIHSS, National Institutes of Health Stroke Scale; TIA, Transient ischemic attack; CHD, Coronary heart disease; IVT, Intravenous thrombolysis.

### RCS regression and subgroup analysis for the clinical outcomes

**Figure 3:**
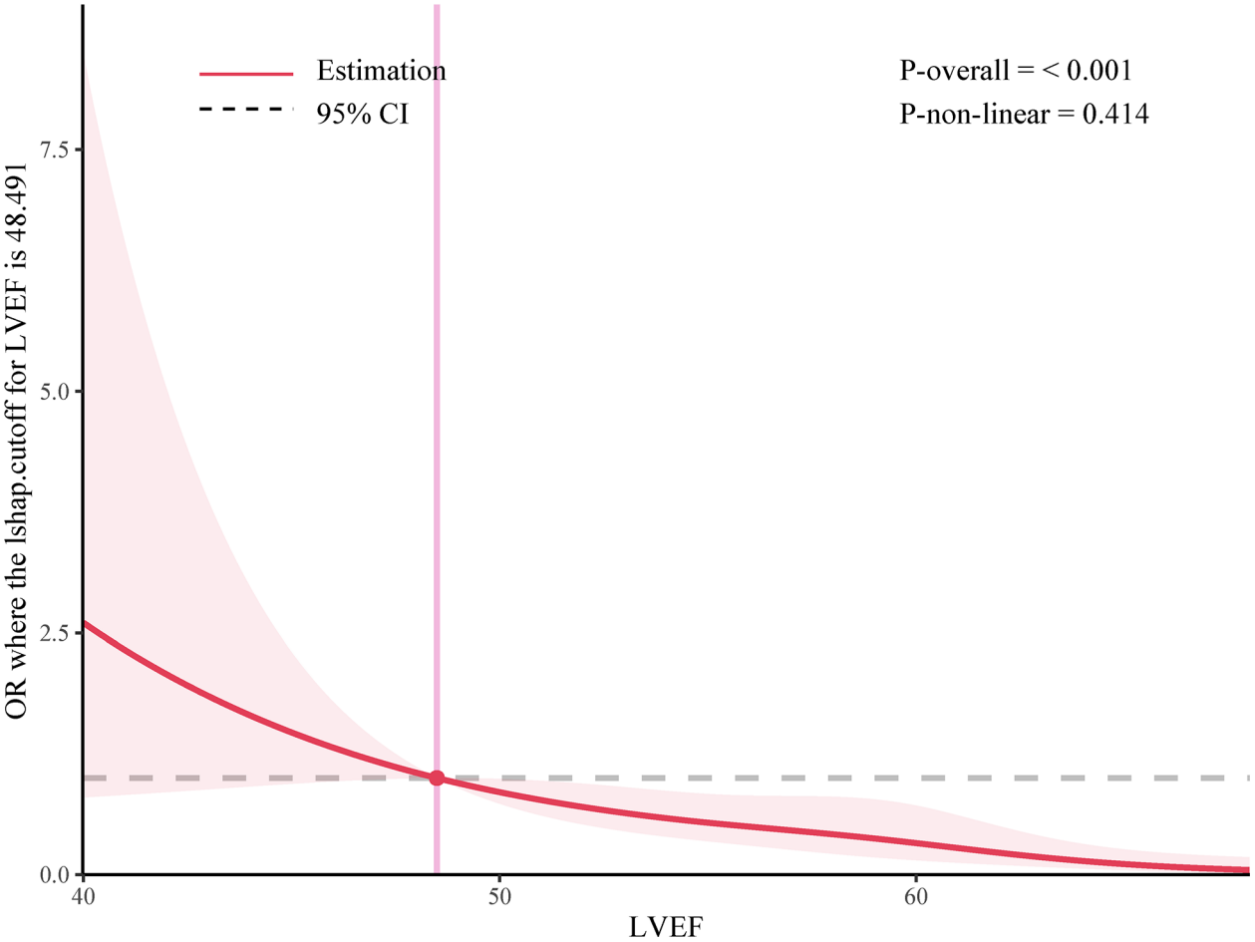
**Adjusted ORs of the 3-month primary outcome according to LVEF levels after IVT.** OR and 95% CI derived from restricted cubic spline regression, with knots placed at the 5th, 35th, 65th, and 95th percentiles of the levels of LVEF after IVT.

**Figure 4:**
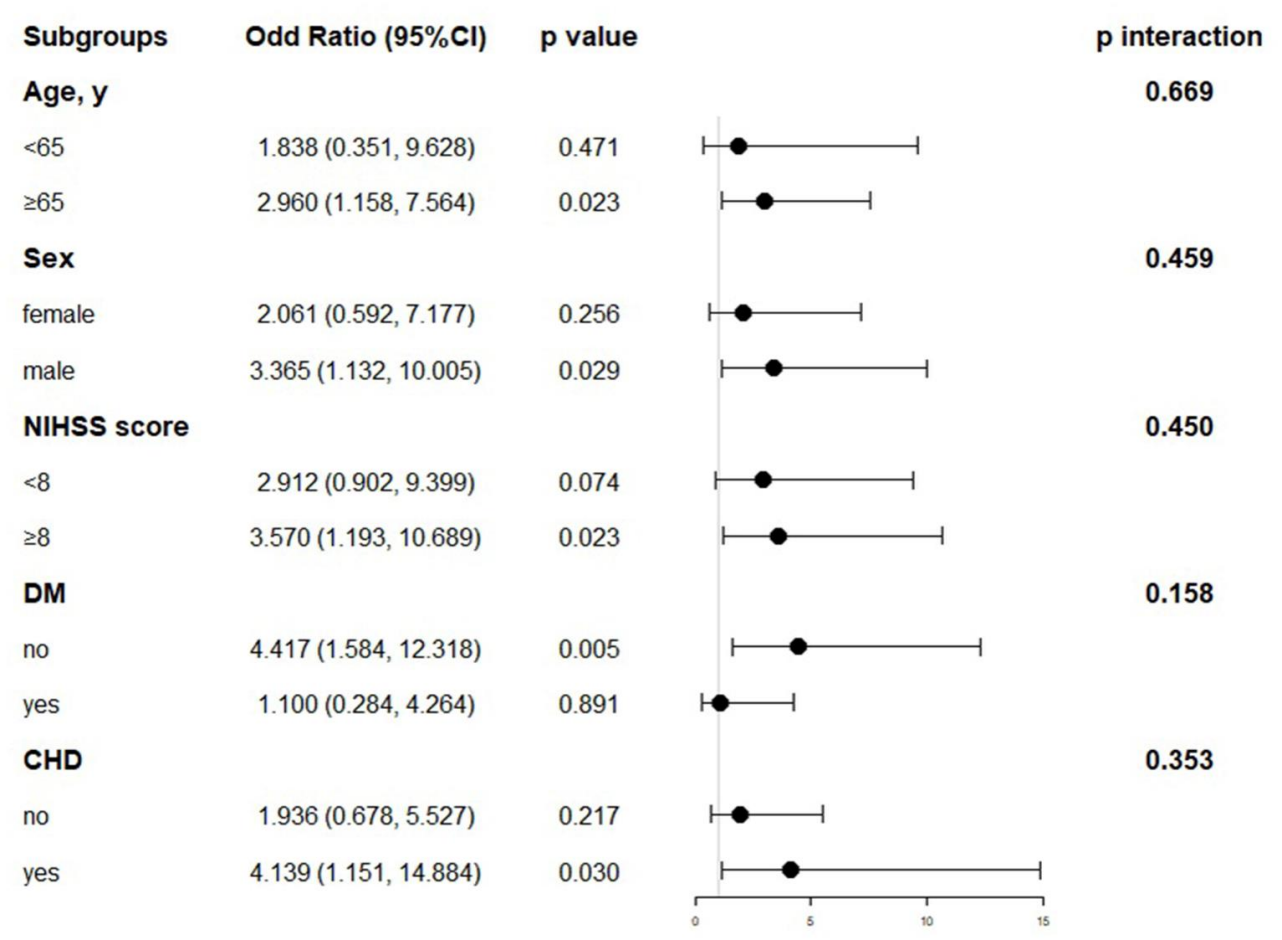
**Subgroup analyses for poor outcomes by LVEF after IVT.** NIHSS, National Institutes of Health Stroke Scale; DM, Diabetes mellitus; CHD, Coronary heart disease.

In the further multivariable logistic regression model with RCS analysis of AIS patients based on LVEF stratified, the proportion of patients achieving good functional outcomes at 90 days significantly decreased with greater degree of LVSD (P for nonlinearity = 0.414; Figure 3). The optimal cutoff values of the LVEF after rt-PA treatment for predicting 90-days poorer functional outcomes were 0.48 (Figure 3). The results of subgroup analyses classified by age (< 65 or ≥ 65 years), sex (female or male), NIHSS on arrival (< 8 or ≥ 8 scores), diabetes mellitus and coronary heart disease are depicted in Figure 4. Obviously, reduced LVEF levels after IVT were discovered to be related with poor outcomes, especially in elderly individuals and higher NIHSS scores (both p=0.023), male (p=0.029), and coronary heart disease (p=0.030).

## DISCUSSION

In this prospective interventional cohort study, we explored the relationship between LVSD and prognosis of AIS patients with 273 cases from the Hefei Hospital Affiliated to Anhui Medical University. The main light of our findings was as follows: (1) The reduced LVEF was significantly associated with END, in-hospital mortality and poor outcomes at 90 days, even after adjusting for sex, age and other comorbidities. (2) RCS analysis showed a negative linear dose-response association between LVEF and functional outcomes at 90 days. (3) The optimal cutoff values of the LVEF after rt-PA treatment for predicting 90-days poorer functional outcomes were 0.48.

Despite the continuous updating of guidelines and widespread promotion of endovascular therapy, intravenous thrombolysis is considered as a preferred and fundamental care for AIS patients who meet certain time-window criteria. Previous studies have reported a correlation between heart failure and cerebral ischemic lesions [20, 21], which indicates a common pathophysiological mechanism underlying AIS and cardiac dysfunction. Data showed that the prevalence of LVSD in AIS patients is as high as 22% [22]. Surprisingly, the majority of contemporary trials and IVT registries did not report or focus on baseline data on cardiac diseases [23–25]. Therefore, few evidence are available about the neurofunctional predictive role of LVSD or heart failure in AIS patients; besides, most studies involved those patients who have not been treated with IVT [26–28].

Studies related to the effect of LVSD or heart failure on functional outcomes and mortality in AIS patients who underwent IVT showed conflicting results. Wahlgren et al. reported that a history of cardiac failure was associated with a 36% increased risk of 90-day mortality [29]. However, recall records of heart failure may underestimate the actual clinical presence of LVSD [30]. In our cohort, 23% of AIS patients had LVSD confirmed by echocardiography vs only 10% with clinical diagnosis of heart failure as determined from self-report of hospital records. While the study by Wei et al. suggested that LVEF<40% was only able to influence mortality, but not neurological outcomes (disability) in AIS patients undergoing IVT [7]. The negative result of the latter study may be attributed to the inclusion of relatively severe heart failure patients (2.4% patients with LVEF<50%), compared with our cohort of AIS patients with LVSD (15.0% patients with LVEF<50%). Therefore, in our view, these inconsistent results may be partially due to different definitions of heart failure and categories of LVEF levels. In the current study, we evaluated the association of LVSD with clinical outcomes in AIS patients against the latest international guidelines for heart failure [31]. Our findings were consistent with a recent study that had similar baseline LVEF characteristics and reported adverse functional outcomes at 90 days in AIS patients treated with IV thrombolysis [32]. In contrast, we added subgroup analysis to improve the stability of the results, and found the best LVEF predictors (48%) of adverse neurological outcomes through restricted cubic spline analysis.

Moreover, previous studies have shown that post-thrombolysis early neurological function changes, especially END, could lead to prolonged hospitalization and severe poor prognosis [33, 34]. In addition to previously identified risk factors, including diabetes [18], NIHSS on admission [35], and systolic blood pressure [36] associated with END after IVT, our study also found that LVSD patients were more prone to END and were closely associated with a poor 90 days functional prognosis.

The pathophysiological mechanism underlying the association between LVSD and poor outcomes in AIS patients was unclear and may be multifactorial. A reasonable hypothesis is that LVSD has a bidirectional effect on both the brain and heart [37, 38]. Although IVT helps to improve cerebral reperfusion, our findings instead support the phenomenon that reduced LVEF may still lead to poor cerebral perfusion, which possibly correlates with decreased brain autoregulation function and network structure after stroke onset [39]. Furthermore, other possible mechanisms for brain-heart interaction include sympathetic and parasympathetic regulation [40], neuroendocrine factors surge [41], and immune and inflammation response [42], negatively impacting the neurorehabilitation process and thus functional outcomes at 90 days. Furthermore, an increase in NIHSS values indicated the extent of cardiovascular autonomic control dysfunction [43]. In this way, either autonomic changes in the brain or heart may put AIS patients at an increased risk of cardiovascular complications and poor prognosis.

Interestingly, after adjusting for relevant confounding variables in the multivariate analysis, apart from LVSD, diabetes mellitus (DM) remained another significant risk factor related with poor outcomes in the current study. This finding was consistent with several prior studies that reported negative outcomes at 90-day in AIS patients treated with IVT or ET [44, 45]. Of note, DM or hyperglycemia was strongly associated with an increased risk of sICH after IVT [46]. A reasonable explanation was that vascular endothelial cell dysfunction and tissue acidosis as a result of central energy metabolism disruption lead to increased blood-brain barrier (BBB) permeability, resulting in cerebral edema, thereby increasing the risk of intracranial hemorrhage [47]. Of note, lower blood glucose levels could also induce sICH after IVT [48, 49]. While Zhang et al. reported that the effective rate after 24h IV was obviously when maintaining blood within the range of 7.0~9.0 mmol/L [48]. Moreover, American Heart Association (AHA) / American Stroke Association (ASA) guidelines suggested that stroke patients with co-existing DM may benefit most from IV time window controlling within 3 hours [50].

Identifying factors that evaluate the effectiveness of thrombolysis in AIS patients may help clinicians predict outcomes, guide early treatment decisions, and inform the development of new interventions. We provide greater insights to this field based on current prospective cohort studies that found a link between readily available clinical indicators of LVEF and the prognosis of AIS patients undergoing IVT. However, some limitations of this study still need to be addressed. Firstly, due to the limited time-window for IVT, LVEF measurement may be inconsistent if performed before as compared to after the stroke events. Secondly, cardiac assessment and echocardiogram at baseline were not always conducted by the same cardiologist, and the lack of validation of heart structure, vascular status, or serological markers (NT pro-BNP) may reduce statistical efficacy. Thirdly, this study did not collect detailed follow-up treatment information, such as prescribed doses of antiplatelet, anticoagulant, or antihypertensive agents, which confounding factors may affect clinical prognosis.

## CONCLUSIONS

Taken together, our findings indicated that LVSD was a stronger predictor of 90-day poor functional outcomes in AIS patients after IVT, suggesting the need to monitor and optimize LVEF in stroke management.
